# Advancing inclusive brain health and dementia care for people with intellectual and developmental disabilities: a public health framework

**DOI:** 10.1093/geront/gnaf222

**Published:** 2025-10-01

**Authors:** Beth Marks, Jasmina Sisirak, Matthew P Janicki, Kathryn P Service, Karen Watchman

**Affiliations:** Department of Disability and Human Development, University of Illinois Chicago, Chicago, Illinois, United States; Department of Disability and Human Development, University of Illinois Chicago, Chicago, Illinois, United States; Department of Disability and Human Development, University of Illinois Chicago, Chicago, Illinois, United States; National Task Group on Intellectual Disabilities and Dementia Practices, Rockport, Maine, United States; National Task Group on Intellectual Disabilities and Dementia Practices, Rockport, Maine, United States; Faculty of Health Sciences and Sport, University of Stirling, Scotland, UK

**Keywords:** Dementia, Developmental disability, Disability, Health equity, Intellectual disability, Structural and social determinants of health

## Abstract

Adults with intellectual and developmental disabilities (IDD) face disproportionately high rates of chronic conditions, including an elevated risk for dementia. Yet access to appropriate brain health promotion and dementia care remains limited due to stigma, underdiagnosis, misdiagnosis, and systemic barriers. This article presents the Healthy Brain Initiative for People with Intellectual and Developmental Disabilities (HBI-PwIDD), which aims to (1) raise awareness of brain health and support health-promoting approaches for people with IDD experiencing Alzheimer’s disease and related dementias; (2) build interprofessional partnerships to develop an inclusive, competent workforce; and (3) strengthen engagement of people with IDD and their supporters in accessing quality healthcare and improving outcomes. Applying a disability intersectionality lens, we integrate brain health promotion with dementia-capable services, emphasizing the critical role of disability-inclusive public health planning. We highlight person-centered approaches grounded in legal and human rights principles that provide access to brain health care and community-based supports. Finally, we discuss how tailored public health messaging and evidence-based workforce strategies can advance national and state brain health and dementia plans and improve equity in care for individuals with IDD. This paper illustrates how integrating disability-inclusive practices within public health systems can promote inclusive aging, improve dementia-related outcomes, and guide gerontologists in building inclusive, life course–oriented models of care.

Adults with intellectual and developmental disabilities (IDD) (see Table 1: Glossary of Terms) encounter significant barriers to brain health promotion and dementia diagnosis, treatment, and care. Despite growing emphasis on healthy aging, inclusive approaches to brain health and access to appropriate and acceptable brain health resources remain scarce for people with IDD (Philip, 2024). Centering people with IDD in brain health promotion and dementia-capable care initiatives offers gerontologists an opportunity to apply an intersectional, public health, and disability-affirming lens to a community historically excluded from aging discourse. Leveraging the evidence-based HealthMatters™ Program and the CDC Healthy Brain Initiative (HBI) Road Map, this article offers actionable strategies for inclusive brain health and dementia care for people with IDD across the lifespan. Gerontologists can use these strategies to “bridge” traditionally siloed domains of aging and disability to advance equity, improve workforce development, and strengthen care systems for all aging populations (Heller and Putnam, 2025) .

**Table 1.** Glossary of terms.

Acronym	Full term
**ADRD**	*Alzheimer’s Disease and Related Dementias*
**BRFSS**	*Behavioral Risk Factor Surveillance System*
**CDC**	*Centers for Disease Control*
**CMS**	*Centers for Medicare & Medicaid Services*
**COPE**	*Care of Older Persons in their Environment*
**DD**	*Developmental Disabilities*
**GWEP**	*Geriatrics Workforce Enhancement Program*
**HBI-PwIDD**	*Healthy Brain Initiative for People with Intellectual and Developmental Disabilities*
**HMP**	*HealthMatters™ Program*
**ID**	*Intellectual Disabilities*
**IDD**	*Intellectual and Developmental Disabilities*
**LHD**	*Local Health Department*
**MCI**	*Mild Cognitive Impairment*
**NACCHO**	*National Association of County and City Health Officials*
**NACDD**	*National Association of Chronic Disease Directors*
**NAPA**	*National Alzheimer’s Project Act*
**NTG**	*National Task Group on Intellectual Disabilities and Dementia Practices*
**PCA**	*Person-Centered Approach*
**S/SDoH**	*Structural and Social Determinants of Health*
**SNAP-Ed**	*Supplemental Nutrition Assistance Program Education*
**UCEDD**	*University Center for Excellence in Developmental Disabilities*
**WHO**	*World Health Organization*

**Table 2.** Brain Health and Dementia Care for People with IDD

Domains	Strategies
1. Strengthen partnerships and policies	Ensure human rights for brain health and dementia care for people with IDD. Advocate for multi-sectoral conversations, including people with IDD and supports and disability, aging, and public health organizations. Disseminate policies for brain health, dementia care, and workforce development to bolster funding, resources, and legislative support for adults with IDD living with dementia (Heller et al., 2018). Link providers with local and state health departments, US Department of Agriculture Cooperative Extension System, SNAP-Ed, and regional parks and recreation departments.
2. Measure, evaluate, and use data	Include people with IDD in national and state surveys. Consider brain health a state of physical, mental, and social well-being across all measurements to address physical and mental health, lack of accessible services, and S/SDOH disparities. Measure and evaluate the impact of accessible models of brain health and dementia care on the needs of individuals with IDD with cognitive changes to promote mobility, engagement, and health outcomes. Provide training hubs and technical support for data-driven healthcare for people with IDD and their supports and a competent workforce with advocacy skills, assistive technology, and disability resources.
3. Build a diverse and skilled workforce	Include people with IDD in workforce development activities with early adopters for early diagnosis, specialized care, and caregiver support to meet the brain health and dementia care needs of individuals with IDD. Train and support formal and informal workforce on aging concerns, chronic conditions and SDOH, intersectional healthcare training, public health collaboration, and disability rights to address the following: 1) staff shortages, technological training, and uniform training standards with systems that assess workforce requirements (Surr et al., 2020); 2) one million individuals (often unpaid family caregivers) aged 60 or older supporting an adult with IDD (Heller et al., 2018); and 3) information to “Know Your Human and Legal Rights” for brain health promotion, dementia care for early screening, diagnosis, and treatment for people with IDD to live, learn, work, play, and love in their communities. Implement a National Plan to Address Alzheimer's Disease and Other Dementias with policies and practices to improve service access for adults with IDD, including those with Down syndrome (Janicki et al., 2016). Connect state-level activities under federally mandated plans, including plans on aging (required by the Older Americans Act) and state plans on developmental disabilities (mandated by Developmental Disabilities Assistance and Bill of Rights Act of 2000) (Administration for ­Community Living, 2000).
4. Engage and educate the public	Coordinate local, state, and national efforts to integrate brain health and dementia care for people with IDD and their caregivers across all public health initiatives. Ensure and provide accessible public service messaging campaigns on websites, public materials, and public service ­announcements for brain health awareness inclusive of people with IDD. Integrate health justice approach to incorporate the complex ways in which disability, class, ethnicity, gender, race, and sexual orientation intersect and create unique, compounded barriers to healthcare for multiple marginalized populations, including African Americans, Indigenous Americans, and Hispanic individuals as well as individuals with IDD (Dorsey Holliman et al., 2023).

As a multidimensional public health priority, promoting brain health across the life course for people with IDD can allow people to “… realize their abilities and optimize their cognitive, emotional, psychological, and behavioral functioning to cope with life situations” (WHO, 2023). The WHO’s definition offers a holistic, disability-affirming lens that supports inclusive brain health promotion, risk reduction, early detection, and dementia-capable care. Integrating the WHO’s definition with the CDC’s definition that focuses on the ability to “remember, learn, play, concentrate, understand and maintain a clear, active mind” (CDC, 2023) broadens the focus from a normative cognitive performance to incorporate diverse ways in which people with IDD, including neurodivergent individuals, think, express, and maintain brain health across the lifespan. Together, these definitions support a life course, rights-based approach that promotes self-determination, embraces neurodiversity, and advances person-centered care to ensure the inclusion of people with IDD in the U.S. public health infrastructure.

The Healthy Brain Initiative for People with Intellectual and Developmental Disabilities (HBI-PwIDD) supports individuals with IDD and their caregivers in accessing culturally appropriate brain health and dementia care across healthcare, public health sectors, and research. Identifying and utilizing key elements of brain health can increase the visibility of people with IDD within public health initiatives. The HBI-PwIDD is led by the HealthMatters™ Program (HMP) at the University of ­Illinois Chicago, in partnership with the National Task Group on Intellectual Disabilities and Dementia Practices (NTG), and the ENGAGE-IL HRSA Geriatrics Workforce Enhancement Program (GWEP). HBI-PwIDD is one of three Component B projects funded by the Centers for Disease Control and Prevention’s (CDC) National Healthy Brain Initiative (2020–2025) and leverages the State and Local Road Map for Public Health 2023–2027 (HBI Road Map). HBI-PwIDD aims to do the following: (1) raise awareness of brain health and early detection and treatment for dementia for people with IDD, (2) strengthen interprofessional partnerships to build a skilled workforce for addressing brain health and dementia, and (3) provide brain health and dementia care resources for people with IDD and their supports to ensure brain health and dementia-capable care for people with IDD within local and state public health departments. By aligning gerontology, public health, and disability networks, this article presents strategies to enhance brain health and dementia care for people with IDD at every stage of life.

## Current status of brain health and dementia care for people with IDD

Intellectual and developmental disabilities (IDD) are lifelong conditions that originate before the age of 22 years and affect cognitive, physical, or adaptive functioning. Common examples include Down syndrome, autism spectrum disorder, cerebral palsy, fetal alcohol spectrum disorders, and Fragile X syndrome. These conditions vary widely but often require long-term support across the lifespan. IDD is an umbrella term that can refer to individuals with both intellectual disability (ID) and other developmental disabilities (DDs). ID is a specific condition characterized by limitations in cognitive and adaptive functioning. At the same time, DD is a broader term encompassing a range of lifelong disabilities that can be intellectual, physical, or both (American Association on Intellectual and Developmental Disabilities, 2023).

### People with IDD, brain health, and dementia

Approximately 6.5 million individuals in the United States have an intellectual disability, the most prevalent developmental disability (Special Olympics, 2025). Among adults aged 60 years and older with IDD, about 6% are diagnosed with dementia, compared to 4% in the general population, with incidence rates increasing with age (Heller et al., 2018; Kramarow, 2024). Adults with Down syndrome, a significant subset of people with ID, face a heightened risk of early-onset dementia linked to AD (Head et al., 2012).

The Lancet Commission has identified 14 modifiable risk factors for dementia (Livingston et al., 2024), many of which disproportionately affect people with IDD. These include chronic conditions (e.g., diabetes, cardiovascular disease, obesity), polypharmacy, limited access to healthcare, undetected vision and hearing impairments, and social inequities (García-Domínguez et al., 2020; Krahn et al., 2010; Thomas, 2019). These disparities underscore the urgent need for inclusive, upstream risk-reduction strategies.

As people with IDD live longer, promoting brain health across the lifespan, identifying risk factors, and early detection of age-related neuropathies, including dementias, are essential for healthy aging (Krinsky-McHale & Silverman, 2013). Accessible health promotion, such as physical activity, nutrition, and social engagement, can reduce dementia risk and improve overall well-being (Albert et al., 2020). However, persistent barriers restrict participation, including underfunded systems, exclusionary practices, negative provider perceptions, and limited access to preventive care. A dementia diagnosis often compounds these inequities, further complicating access to appropriate services (Heller et al., 2018).

Addressing these challenges requires a dual approach with proactive upstream strategies and downstream responsive healthcare. Together, these strategies can address the structural and social determinants of health (S/SDoH), which account for 80%–90% of health outcomes (Magnan, 2017). Advancing equitable brain health for people with IDD requires a rights-based, person-centered approach (PCA) that integrates chronic disease management, dementia prevention, and disability inclusion.

### Reframing dementia with a disability lens

Dementia care has traditionally been viewed through a medical lens (Shakespeare et al., 2019). Although individuals with dementia do not always identify as disabled, members of Dementia Alliance International actively asserted their rights under the United Nations Convention on the Rights of Persons with Disabilities (CRPD) at the WHO’s 2015 First Ministerial Conference on Dementia (WHO, 2015). Their advocacy emphasizes the right to continue living fulfilling lives as active, valued members of their communities (Alzheimer Europe, 2017). This rights-based approach aligns with the longstanding advocacy of people with IDD and their allies, who for over 75 years have fought to live, learn, work, play, and love in their communities rather than be institutionalized (The Arc, 2025). This alignment is critical because it offers a unified, life course–driven vision of inclusion that challenges the historical and ongoing segregation of both people with IDD and those living with dementia.

Dementia advocacy increasingly incorporates the social model of disability and principles of disability justice into dementia care frameworks. As individuals with dementia assert their human and legal rights, they challenge the social and physical barriers that limit autonomy and inclusion. A disability-affirming approach recognizes the intersecting barriers faced by marginalized groups and the systems that reinforce them (Hudson, 2024). This is especially critical for people with IDD, who are disproportionately represented in racially, ethnically, and economically marginalized communities and face compounded inequities rooted in structural barriers (Farrell et al., 2022).

Many national and global guidelines still treat disability as an isolated condition rather than a core part of human identity (Wickenden, 2023). Dementia, which affects people across all cultural, linguistic, and socioeconomic groups, remains deeply stigmatized because of negative societal attitudes and longstanding fears about disability (Siette et al., 2023). Cultural perceptions, communication barriers, and limited awareness delay diagnosis and restrict access to appropriate care and support.

### “Double jeopardy” and “weathering”

People with IDD who develop dementia face compounded barriers, including systemic bias, discriminatory attitudes, and inadequate healthcare that delay early detection and limit access to treatment (Watchman et al., 2017). The intersection of disability with other marginalized identities, such as age, race, ethnicity, socioeconomic status, and immigration status, further shapes how individuals with IDD experience the healthcare system (Wickenden, 2023). One pathway through which systemic inequality impacts brain health is “weathering,” the cumulative impact of chronic stress on the body. Weathering has been linked to compromised brain development, decreased resilience to stress, and increased allostatic load, the physiological wear and tear from prolonged exposure to adversity (Geronimus et al., 2006; Hines et al., 2023). An intersectional approach to dementia care emphasizes upstream prevention through equitable access to screening tools, timely diagnosis, trained providers, and appropriate care (Turan et al., 2019).

Tailoring brain health and dementia initiatives for people with IDD also requires addressing the S/SDoH that shape access and outcomes. People with IDD from racially, ethnically, and culturally marginalized communities often have lower average life expectancies, reflecting the compounded impact of structural racism and ableism (Landes et al., 2022). This “double jeopardy” effect, also described as a “double disadvantage to health,” is a key factor in reduced life expectancy among people with IDD who belong to racial–ethnic minority groups (Farrell et al., 2022).

Among people without IDD, reduced life expectancy compared to White peers is significant: 1.9 years for Asian/Pacific Islanders, 8.5 years for Black Americans, 8.8 years for Hispanic Americans, and 11.8 years for American Indians (Landes et al., 2022). Landes et al. (2022) also noted that the impact of double jeopardy varies by disability type and racial–ethnic group; for example, life expectancy disparities among people with Down syndrome were only evident for Asian/Pacific Islanders.

## Brain health and dementia-capable care for people with IDD

In response to the disproportionate risk of dementia among individuals with IDD, the HBI-PwIDD promotes brain health promotion across the lifespan and advances dementia-capable care for people with IDD and their supports. Leveraging the 2023 HBI Road Map, HBI-PwIDD integrates disability considerations into national public health strategies for brain health and dementia care (Alzheimer’s Association and Centers for Disease Control and Prevention, 2023). It operationalizes the Road Map’s four core domains: (1) strengthen partnerships and policies; (2) measure, evaluate, and utilize data; (3) build a diverse and skilled workforce; and (4) engage and educate the public to provide accessible brain health and dementia-care resources for people with IDD.

### Strengthen partnerships and policies

With cross-sector partnerships, HBI-PwIDD provides technical assistance to organizations such as Alzheimer’s Association Chapters, State Developmental Disabilities Councils, and Special Olympics to advance integrated public health and policy agendas for brain health promotion and dementia-capable care initiatives. Collaborative efforts across healthcare, public health, and community organizations promote equity-informed, IDD-inclusive frameworks. Challenges remain with approximately 60% of adults with IDD in the U.S. outside state developmental disability service systems, rendering them invisible primarily to public health surveillance and policy efforts (Rosencrans et al., 2021). This “hidden majority” faces increased risk of diagnostic overshadowing, where symptoms of dementia are misattributed to pre-existing disabilities.

HBI-PwIDD expands access by including people with IDD in evidence-based dementia care initiatives. For example, collaboration with a COPE (Care of Older Persons in their Environment) initiative developed strategies to raise awareness of caregiving needs for people with IDD and their caregivers (Oakwood Creative Care, 2025). Partnerships with ENGAGE-IL, HRSA Geriatrics Workforce Enhancement Program, and University Centers for Excellence in Developmental Disabilities (UCEDDs) enhance public health competency by disseminating universal design brain health resources such as screening tools and training materials tailored for individuals with IDD.

National Task Group (NTG) policy advocacy promoted inclusion of people with IDD in dementia risk-reduction efforts, including within the National Alzheimer’s Project Act (NAPA) and the Federal Advisory Council on Alzheimer’s Research, Care, and Services. NTG and the HealthMatters™ Program (HMP) participated in the 2nd International Summit on Intellectual Disability and Dementia (Janicki et al., 2025), highlighting global perspectives on human rights, neurodiversity, and the role of S/SDoH in brain health.

### Measure, evaluate, and utilize data

Despite well-documented disparities, data on dementia among people with IDD remain limited. The lack of population-based data limits precision-based personalized preventive, diagnostic services, interventions, and care for people with IDD (August & Gewirtz, 2019; Havercamp & Scott, 2015), hindering equitable resource allocation and effective policy responses (Krahn et al., 2010). Significant challenges include the following: (1) lack of consistent IDD identifiers in national surveys; (2) inadequate dementia screening tools for people with cognitive differences; (3) sampling biases in IDD research; and (4) standardized methods for assessing dementia in individuals with IDD (Hatton et al., 2015).

With the lack of inclusion of people with IDD in the Behavioral Risk Factor Surveillance System (BRFSS) (Centers for Disease Control and Prevention (CDC), 2018), addressing health disparities and allocating resources for dementia care remains unstable. For example, timely, evidence-based screening and diagnosis of cognitive changes and dementia with current screening tools is inadequate for individuals with IDD (Krinsky-McHale & Silverman, 2013). Many dementia assessments do not distinguish between pre-existing cognitive disabilities and new-onset dementia, leading to diagnostic inaccuracies (Zeilinger et al., 2013). Factors like age, education level, language, and cultural background can further complicate reliable dementia diagnosis for people with IDD (Czerwinski-Alley et al., 2024).

To address the challenges related to screening and diagnosing cognitive changes among people with IDD, NTG’s publication, *Examining Adults with Neuroatypical Conditions for Mild Cognitive Impairments (MCI)/Dementia During Cognitive Impairment Assessments*, provides recommendations to include adaptations of assessment practices to accommodate neuroatypical conditions (Janicki et al., 2022). This report guides clinicians in developing educational packets about neuroatypical conditions, detecting, and diagnosing MCI or dementia. Research on evidence-based information assessing later-life adult cognitive diseases and planning post-diagnostic care for people with IDD remains critical.

### Build a diverse and skilled workforce

A diverse and skilled workforce is essential to reducing dementia risk and improving health outcomes for individuals with IDD. HBI-PwIDD expands workforce capacity to reduce stigma, promote brain health, and deliver culturally competent dementia care in alignment with national public health strategies. Using a multisectoral approach grounded in where people with IDD live, learn, work, play, and love, HBI-PwIDD incorporates the S/SDoH that shapes brain health within its cornerstone initiative, the HealthMatters™ Program (HMP). HMP is a 12-week behavioral lifestyle intervention designed to promote brain and heart health for people with IDD and their supports (Marks et al., 2013; Marks, Sisirak, Chang, & Murphy, 2019). HMP includes three components that meet the Administration for Community Living’s Title III-D Highest Tier Evidence-Based Health Promotion/Disease Prevention Programs (ncoa.org/article/evidence-based-program-healthmatters-program): (1) a Certified Instructor Workshop Webinar; (2) Health Matters: The Exercise and Nutrition Health Education Curriculum for People with IDD; and (3) a 12-Week HMP for PwIDD 3 times/week. Studies demonstrate improved physical and psychosocial health, nutrition and physical activity knowledge, fitness, and self-efficacy among PwIDD and caregivers (Marks et al., 2013). The program’s 36 instructional lessons and 23 continuing education lessons incorporate plain language, self-determination, and advocacy skills with input from people with IDD, families, and both licensed and unlicensed providers.

Over 800 instructors from 175 organizations across 37 states have been certified through HBI-PwIDD, expanding the reach of brain health promotion across community and clinical settings (HealthMatters Program, 2024). HMP’s trainings, curriculum, health advocacy resources, and fact sheets incorporate universal design principles and address S/SDoH for brain health, such as stigma, to enhance healthcare access for people with IDD. Through HBI-PwIDD, the instructor network has grown beyond traditional disability service agencies to include federally funded programs such as the Supplemental Nutrition Assistance Program Education (SNAP-Ed), Area Agencies on Aging (AAA), and the USDA’s Cooperative Extension System. SNAP-Ed and AAA have adapted HMP to reach individuals with IDD and their communities through local health promotion efforts. The Cooperative Extension System partners with other community organizations supporting people with IDD and is implementing the HealthMatters Program with local cooperators and collaborators to advance health education, address state and national issues/trends, and respond to local concerns to achieve community health goals.

Developed in partnership with HBI-PwIDD, NTG’s Changing Thinking! Initiative aligns workforce training for Navigators and Practitioners with the Centers for Medicare and Medicaid Services (CMS) GUIDE Model. This initiative builds dementia care competencies for those serving Medicare-eligible people with IDD and enhances diagnostic accuracy, care access, and social supports . HBI-PwIDD collaborates with the two other CDC-funded Component B grantees and supports training within the UsAgainstAlzheimer’s Brain Health Equity Nurse Fellows Program. This program increases understanding of how intersectional identities, cultural values, and systemic barriers shape dementia risk for African American, Latino, American Indian, and Alaska Native communities, including those with IDD. Each cohort of 12 fellows develops skills to identify and respond to the cumulative effects of the intersectionality of multiple identities and structural inequities that impact brain health and dementia care.

Comprehensive, evidence-based workforce education fosters a more responsive and inclusive infrastructure for brain health and dementia care. Investments in workforce development build capacity to address disparities and deliver person-centered, culturally relevant care. Through training, research partnerships, technical assistance, and dissemination strategies, the HBI-PwIDD network, alongside programs like SquarePeg Training (2025), enhances interprofessional competencies and expands access to dementia-related services for people with IDD and their supports.

### Engage and educate the public

The HealthMatters™ Program (HMP) and the National Task Group (NTG) collaborate to deliver accessible educational initiatives through digital platforms and community partnerships. A flagship effort is the Healthy Brain Webinar Series for People with IDD, which featured 28 presentations on brain health, dementia care, caregiver support, nutrition, and healthcare access. The series on YouTube has attracted over 10,000 views, reflecting strong public interest in inclusive brain health education for people with IDD and their supports. Continued access to these webinars and HMP and NTG’s programmatic outreach to families, agencies, and caregivers ensures that people with IDD and their supports receive practical guidance on dementia prevention and care. This initiative underscores the role of digital education in expanding awareness and reducing disparities in brain health knowledge.

HBI-PwIDD partnered with the International Association for Indigenous Aging (IA2) to promote culturally responsive public health messaging. Through talking circles, American Indian and Alaska Native individuals with IDD and their supports shared lived experiences and identified culturally grounded strategies for brain health promotion. These insights guide tailored outreach that aligns with community values and traditions. HBI-PwIDD also collaborates with the Alzheimer’s Disease and Related Dementias (ADRD)/Brain Health Panel, convened by the National Association of Chronic Disease Directors (NACDD), to develop technical assistance tools that expand the capacity of BOLD Public Health programs and state and local health departments to be inclusive of people with IDD.

At a local level, a partnership between HBI-PwIDD, a local health department (LHD), and a community service provider is advancing dementia prevention by increasing physical activity, improving nutrition, strengthening self-advocacy, and reducing social isolation, key factors for lifelong brain health. The initiative fostered an inclusive health culture by integrating the HealthMatters Program (HMP) across schools, community centers, and care facilities, driving lasting change at both individual and organizational levels. In recognition of this achievement, the local health department received the 2025 National Association of County and City Health Officials (NACCHO) Model Practice Award for its initiative to include people with IDD. The project was honored as a national model for advancing inclusive public health through peer engagement, responsive teaching, and community-led strategies.

## The way forward

Looking ahead, a person-centered approach (PCA) can guide the integration of brain health and dementia care for people with IDD (Calatayud et al., 2024). The My Thinker is Working Brain Health Action Plan builds on the National Task Group’s foundational strategy, My Thinker’s Not Working (NTG, 2012), which established a national framework for supporting adults with IDD affected by Alzheimer’s disease and related dementias (ADRD) to remain in their communities and receive high-quality care.

Grounded in PCA, disability rights, and public health best practices, *My Thinker is Working* offers actionable steps and policy guidance to promote brain health and reduce dementia risk among people with IDD. Continued advocacy is essential to ensure the inclusion of people with IDD in national data systems, supporting equitable, evidence-based policymaking and resource allocation. Future efforts can prioritize universally designed programs and assessment tools that reflect the full diversity of populations.

### S/SDoH, disability, and dementia

Recognizing dementia as both a medical condition and a disability expands the scope of care to include S/SDoH, such as stigma, economic instability, and access barriers. WHO’s Global Action Plan on the Public Health Response to Dementia (2017–2025) and the American Public Health Association’s 2020 policy statement emphasize the need for disability-inclusive public health messaging and service delivery (American Public Health Association, 2020). The U.S. National Institutes of Health’s recent designation of people with disabilities as a health disparity population reinforces the importance of tailored research, funding, and policy initiatives to reduce dementia risk for people with IDD (National Institutes of Health, September 26, 2023).

### My thinker is working: brain health in action

The HBI-PwIDD is finalizing a plan for “*My Thinker is Working: Brain Health in Action*,” which includes the following:

1. Early Equitable Access to Care—Address the “double jeopardy” of individuals with IDD and dementia with screenings, accurate diagnoses, and proactive interventions.
2. Integrated Accessible Care Models—Develop brain health and dementia care to meet the needs of people with IDD, reduce caregiver stress, and provide support.
3. Workforce Development and Training—Equip caregivers, healthcare, and public health providers to recognize and manage dementia in neurodiverse communities.
4. Sustainable Public Health Policies—Secure long-term funding, resources, and legislation for brain health and dementia care for individuals with IDD.

By centering the voices and needs of individuals with IDD and their supports for brain health and dementia care, *My Thinker is Working: Brain Health in Action* can support effective brain health and improve the lives of people with IDD.

### Community partnerships for reach, sustainability, and impact

Multi-sectoral community partnerships are essential to improving the reach, impact, and sustainability of brain health and dementia care for individuals with IDD. Applying universal design principles to public health messaging, workforce training, and policy ensures that brain health initiatives are accessible, inclusive, and responsive to the needs of people with IDD (van Corven et al., 2022).

Sustained engagement among healthcare professionals, public health leaders, individuals with IDD, and caregivers creates an ecosystem of support where dementia-related services can be delivered in a timely and equitable manner. At the local, state, and national levels, initiatives that adopt disability-inclusive approaches can enhance brain health promotion and dementia prevention while addressing S/SDoH.

As summarized in Table 2 Brain Health and Dementia Care for People with IDD Table 1, community collaborations can:

- Amplify Public Health Messaging—Universal design in health communication ensures brain health and dementia information reaches individuals with IDD, their supports, and providers in ways that are accessible and engaging.
- Promote Sustainable Policy Change—Community-driven advocacy can shape legislative and policy frameworks that prioritize dementia care within disability services and secure long-term investment in inclusive brain health strategies.
- Strengthen Workforce Training—Dementia-specific skills training for public health and healthcare providers supports early identification, accurate diagnosis, intervention, and delivery of person-centered care.
- Enhance Care and Support Networks—Local partnerships help implement dementia-friendly practices in community settings, increasing service accessibility and improving health and quality-of-life outcomes.

By including these multi-sectoral practices in public health infrastructure, we can ensure that people with IDD who are at risk for or living with dementia receive dignified, equitable, and comprehensive care.

## Conclusion

The collective efforts described in this paper underscore the urgent need for an intersectional, multi-sector approach to reduce dementia risk among adults with IDD. Elevated risks due to chronic conditions, compounded by stigma, underdiagnosis, systemic barriers, and a lack of culturally competent care, necessitate disability-inclusive public health strategies to improve brain health outcomes.

Integrating person-centered, rights-based practices within national and state-level brain health initiatives is essential to address long-standing disparities for everyone. Key strategies include integrated care models, culturally accessible, responsive public health messaging, and sustained investment in workforce development. Through HBI-PwIDD, cross-sector collaboration has (1) delivered the evidence-based HealthMatters™ Program to address modifiable risk factors and improve health behaviors, (2) raised awareness of brain health and dementia care for people with IDD, (3) expanded dementia-capable interprofessional workforce, and (4) strengthened engagement of individuals with IDD and their supports to access brain health and dementia care resources.

Looking ahead, the *My Thinker is Working Brain Health in Action* offers a roadmap for advancing equitable, dignified, and accessible brain health promotion and dementia-capable care for individuals with IDD. Through evidence-based policy reform and systems-level coordination, we can build a more inclusive public health infrastructure that supports brain health across the lifespan and affirms the rights and dignity of people with IDD. Gerontologists can play a pivotal role by advancing research, education, and practice models that include people with IDD and their caregivers, ensuring age-related policies and services are inclusive of this historically marginalized population.

## Supplementary Material

gnaf222_Supplementary_Data

## Data Availability

The authors do not report data, so the pre-registration and data availability requirements are not applicable.
