# Supplementary material for: Advancing inclusive brain health and dementia care for people with intellectual and developmental disabilities: a public health framework
Source: Gerontologist. 2025 Oct 1;65(Suppl 1):S60–7. doi: 10.1093/geront/gnaf222 (PMC12736998; doi:10.1093/geront/gnaf222)
Supplement: gnaf222_Supplementary_Data [file gnaf222_supplementary_data.zip › gnaf222_Supplementary_Data/Table_2_Brain_Health_and_Dementia_Care_for_People_with_IDD.docx]

**Supplementary Material**

**Table 2. Brain Health and Dementia Care for People with IDD**

| Domains | Strategies |
| --- | --- |
| 1. Strengthen partnerships and policies | 1. Ensure human rights for brain health and dementia care for people with IDD. 2. Advocate for multi-sectoral conversations, including people with IDD and supports and disability, aging, and public health organizations. 3. Disseminate policies for brain health, dementia care, and workforce development to bolster funding, resources, and legislative support for adults with IDD living with dementia (Heller et al., 2018; Yee et al., 2018). Link providers with local and state health departments, US Department of Agriculture Cooperative Extension System, SNAP-Ed, and regional parks and recreation departments. |
| 1. Measure, evaluate, and use data | 1. Include people with IDD in national and state surveys. 2. Consider brain health a state of physical, mental, and social well-being across all measurements to address physical and mental health, lack of accessible services, and S/SDOH disparities. 3. Measure and evaluate the impact of accessible models of brain health and dementia care on the needs of individuals with IDD with cognitive changes to promote mobility, engagement, and health outcomes (Matin et al., 2021). 4. Provide training hubs and technical support for data-driven healthcare for people with IDD and their supports and a competent workforce with advocacy skills, assistive technology, and disability resources. |
| 1. Build a diverse and skilled workforce | 1. Include people with IDD in workforce development activities with early adopters for early diagnosis, specialized care, and caregiver support to meet the brain health and dementia care needs of individuals with IDD. 2. Train and support formal and informal workforce on aging concerns, chronic conditions and SDOH, intersectional healthcare training, public health collaboration, and disability rights to address the following: 1) staff shortages, technological training, and uniform training standards with systems that assess workforce requirements (Surr et al., 2020); 2) one million individuals (often unpaid family caregivers) aged 60 or older supporting an adult with IDD (Heller et al., 2018); and 3) information to "Know Your Human and Legal Rights" for brain health promotion, dementia care for early screening, diagnosis, and treatment for people with IDD to live, learn, work, play, and love in their communities. 3. Implement a National Plan to Address Alzheimer's Disease and Other Dementias with policies and practices to improve service access for adults with IDD, including those with Down syndrome (Janicki, 2016). Connect state-level activities under federally mandated plans, including plans on aging (required by the Older Americans Act) and state plans on developmental disabilities (mandated by Developmental Disabilities Assistance and Bill of Rights Act of 2000) (Administration for Community Living, 2000). |
| 1. Engage and educate the public | 1. Coordinate local, state, and national efforts to integrate brain health and dementia care for people with IDD and their caregivers across all public health initiatives. 2. Ensure and provide accessible public service messaging campaigns on websites, public materials, and public service announcements for brain health awareness inclusive of people with IDD. 3. Integrate health justice approach to incorporate the complex ways in which disability, class, ethnicity, gender, race, and sexual orientation intersect and create unique, compounded barriers to healthcare for multiple marginalized populations, including African Americans, Indigenous Americans, and Hispanic individuals as well as individuals with IDD (Dorsey Holliman et al., 2023). |
